# Urticaria as a manifestation of hyperthyroidism

**DOI:** 10.1002/ccr3.3620

**Published:** 2021-05-05

**Authors:** Nicholas Womack, Edward Jude

**Affiliations:** ^1^ Tameside and Glossop Integrated Care NHS Foundation Trust Ashton‐Under‐Lyne UK

**Keywords:** carbimazole, hyperthyroidism, urticaria

## Abstract

In clinically euthyroid patients presenting with urticaria, a trial period of withholding antithyroid medications can be exercised. In clinically hyperthyroid patients, antithyroid medications may be stopped with close observation of response.

## Graphical abstract

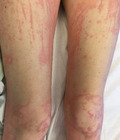

## 1 BACKGROUND

Urticaria is a common, pruritic skin condition with multiple causes and can have a significant impact on quality of life. In this paper, we present a case of acute urticaria secondary to hyperthyroidism. This link appears to be due to the autoimmune thyroid cascade (particularly IgE antithyroid peroxidase [TPO] antibodies) sensitizing mast cells for activation and histamine release. Although antihistamines and steroids are first‐line treatment, identifying the “causative” factor is not easy in hyperthyroid patients due to the independent links urticaria shares with both carbimazole and thyrotoxicosis.

Urticaria consists of blanchable, erythematous “wheals” secondary to vasoactive mediators, predominantly histamine release from mast cells. Wheals are usually transient, disappearing from one aspect of the body and reappearing in new areas, and almost always pruritic. Acute urticaria refers to a presentation of <6 weeks; otherwise, the term chronic urticaria is used. Most cases are idiopathic, but known triggers include drugs, infections, foods, and even certain physical factors (such as cold, heat, and sunlight).^1^

Hyperthyroidism results from an excess state of thyroid hormones caused by excess release and increased synthesis from the thyroid gland, or less commonly, from extrathyroidal sources. Excess thyroid release is often stimulated by specific antibodies, including antithyroid peroxidase (TPO), antithyroglobulin (Tg), and antithyroid‐stimulating hormone receptor (TSHR) antibodies.^2^ Graves' disease, the most common cause of hyperthyroidism, is an example of an autoimmune disease where thyroid‐stimulating antibodies activate thyroid‐stimulating hormone receptors, leading to excess hormone release from the thyroid gland.^3^

Urticaria and autoimmune hyperthyroidism appear to share an aetiological relationship, although the exact mechanism remains ambiguous.^4^ In this report, we discuss a new case of urticaria presenting itself as a manifestation of hyperthyroidism, the pathophysiology to this link is explored and management strategies are discussed.

## 2 CASE PRESENTATION

**FIGURE 1:**
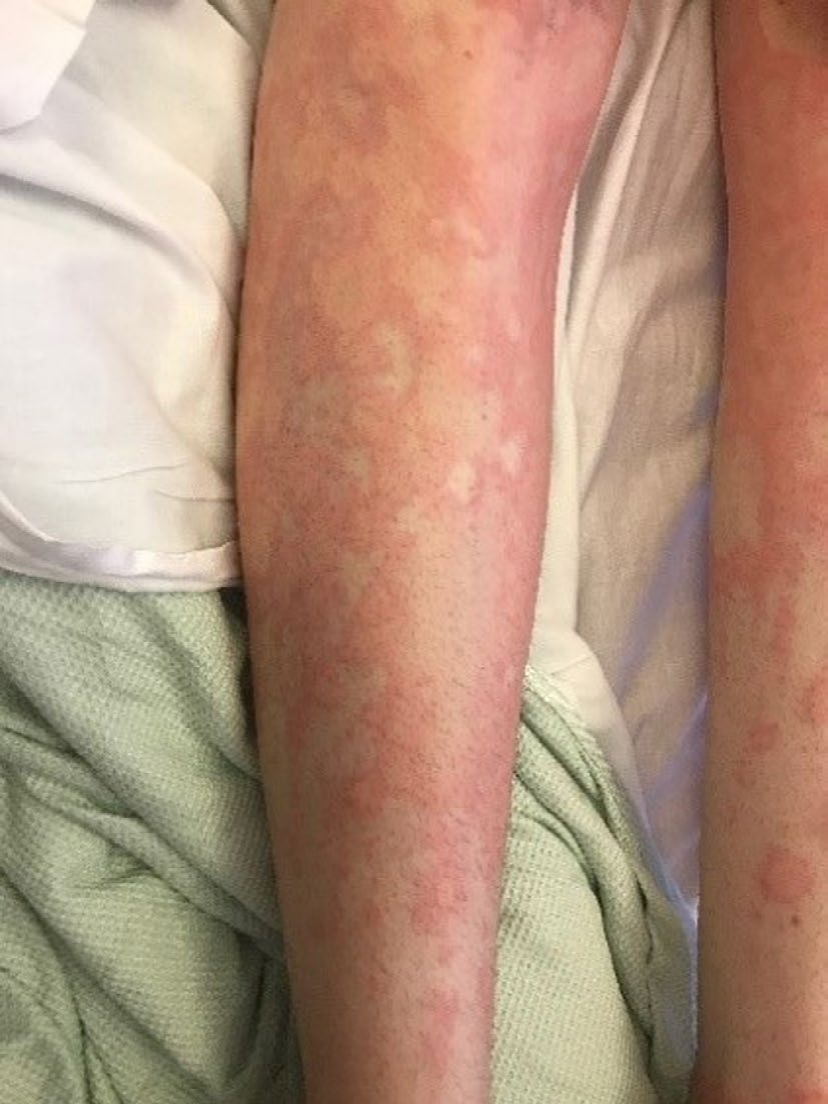
Patient presented with acute urticaria

**FIGURE 2:**
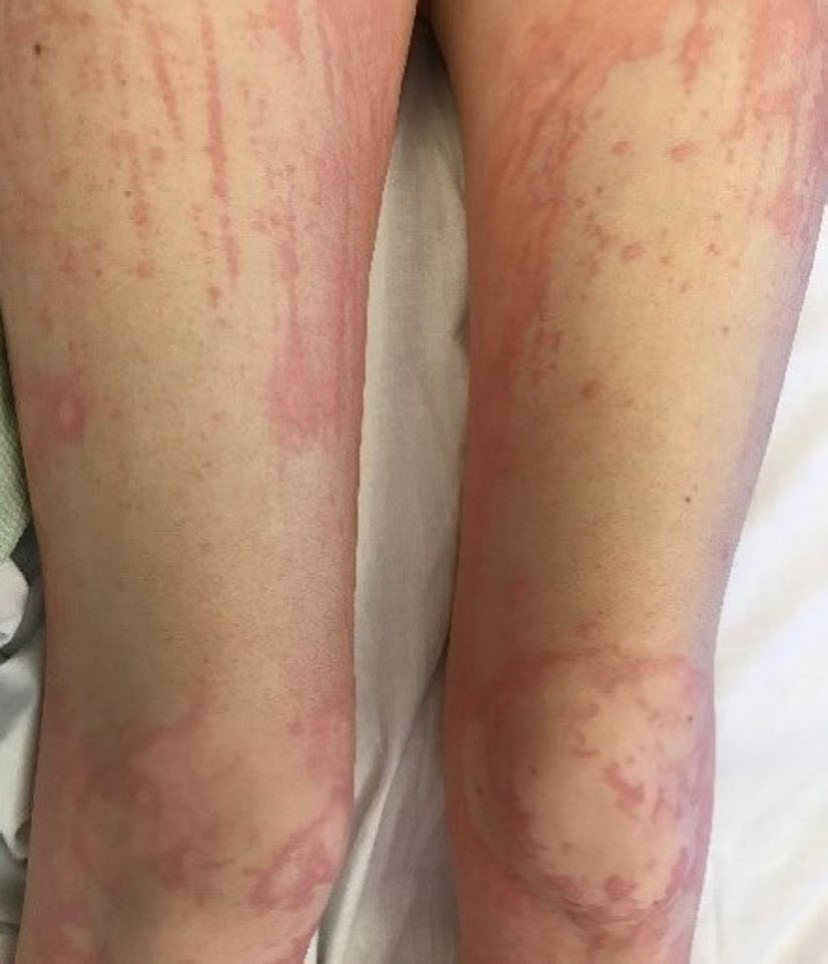
Patient presented with acute urticaria

A 23‐year‐old female patient with a recent diagnosis of Graves' disease presented to her GP with an urticarial rash localized to her neck. She was prescribed a course of cetirizine and prednisolone. Shortly after, she presented to the emergency department with a spreading urticarial rash extending to her neck, stomach, chest, back, genitals, upper and lower legs, consisting of “extensive excoriations and dermatographia, with blanching and wheals” (Figures 1 and 2). She had given birth to her first child as recently as 4 months ago and the only other medication she took on a regular basis was carbimazole, which was started 1 month prior to her presentation. She had no family history of any autoimmune disorders.

Investigations revealed normal inflammatory markers (CRP 2 mg/L, WBC 9.3 × 10^9^/L), and her renal and liver function tests all remained within normal limits. No clinical findings were evident on examination of her chest and abdomen, and no focal neurology was elicited. Her FT4 2 weeks prior to admission was 38.4 pmol/L, with a TSH of 0.01 mU/L, although her FT4 on the day of admission was normal at 10.7 pmol/L. She was treated with intravenous chlorphenamine in the emergency department then admitted for observation and referred to the endocrinologist.

Over the next 48 hours, she had a further exacerbation of her rash which now resulted in lip swelling and she was started on intravenous hydrocortisone and oral loratadine. Her carbimazole was stopped due to concerns it may be the cause of the rash. She was reviewed the next day and an urgent dermatology opinion was sought, while a full autoimmune screen was sent (*all negative*, Table 1). Urinalysis was also negative. Thyroid peroxidase antibody (anti‐TPO) levels were raised at >1000 iU/mL, and thyroid receptor antibodies were unremarkable (0.30 iU/L).

**TABLE 1:** Autoimmune screening tests

Antibody	Result
Complement (C3)	1.26 g/L (negative)
Complement (C4)	0.14 g/L (negative)
ANCA (Antineutrophil cytoplasmic antibody)	<0.2 iU/mL (negative)
Rheumatoid factor	17.6 iU/mL (negative)
ANA (Antinuclear antibody)	Negative
Anticardiolipin	<1.6 GPLU/mL (negative)

After dermatology review, the patient was started on oral chlorphenamine (at triple the normal licensed dose), topical menthol 1% cream and her steroid dose was gradually reduced. After 5 days of treatment, her urticaria was limited to her legs and scalp, and she had symptomatically improved.

## 3 OUTCOME AND FOLLOW‐UP

Once the patient's symptoms had improved, she was discharged having stayed in hospital for a total of 1 week. She had an ultrasound of her thyroid 2 weeks after discharge which showed diffuse thyroiditis along with a right U2 thyroid nodule. She was discharged without any further carbimazole and followed up in endocrine clinics to monitor thyroid function and to potentially commence propylthiouracil.

## 4 DISCUSSION

The lifetime prevalence of acute urticaria is estimated to be 15%‐23% in adults and up to 14.5% in children.^5^ The detriment to quality of life is severe, and applicable to social, recreational, vocational, and emotional aspects of life.^6^, ^7^ Management consists of high dose antihistamines along with steroid use and avoidance of causative factors.^8^

In rare circumstances, urticaria can be the presenting feature of hyperthyroidism. Rothfield^9^ reviewed 108 cases of hyperthyroidism and found two cases of urticaria (1.85%) that varied with the degree of hyperthyroidism. Pruritis was also found in a number of patients, and severity varied along with thyroid state in five out of the 108 cases (4.63%).

The mechanism linking urticaria and hyperthyroidism is not clearly understood. Chronic urticaria is thought to be triggered by activation and degranulation of mast cells. Although IgG antithyroid auto‐antibodies do not directly activate mast cells, they are postulated to make them more susceptible to activation by other mediators.^10^ In this case, thyroid peroxidase antibodies were >1000 iU/mL. Another proposed theory is that IgE anti‐TPO antibodies directly bind to mast cells to cause autoallergic mast cell degranulation and activation.^11^ Furthermore, omalizumab, an anti‐IgE therapy, provides particularly good symptomatic improvement in patients with urticaria and positive IgE anti‐TPO antibodies.^12^

Treatment for hyperthyroidism consists of medications, surgery, or radioiodine. The currently licensed medications for hyperthyroidism include carbimazole (converted to methimazole) and propylthiouracil, both of which reduce the conversion of thyroxine (T4) to its active form, triiodothyronine (T3).^13^, ^14^ Specifically, methimazole and propylthiouracil impede iodination of tyrosine residues in thyroglobulin, an important step in conversion of T4 to T3. Propylthiouracil also inhibits peripheral conversion of T4 to T3, although the therapeutic effect of this is unclear.^13^

Carbimazole itself is also a known cause of rash and urticaria.^15^, ^16^ A review of the yellow card scheme between 1981 and 2003 found rashes to be the 4th most common side effect for carbimazole, behind neutropenia, hepatobiliary disorders, and agranulocytosis. Interestingly, angioedema is also a recognized side effect of carbimazole, which could indicate that carbimazole was the underlying cause in our case.^17^ Propylthiouracil, the lesser used medication for hyperthyroidism, has a number of adverse effects including urticaria and other skin reactions; skin eruptions occur in 4%‐6% of patients.^18^

In patients that are otherwise clinically euthyroid, a suitable strategy would be to withhold carbimazole (or propylthiouracil) when an urticarial rash first develops. But the correct action in patients with uncontrolled hyperthyroidism is less clear, as withholding antithyroid drugs could result in an exacerbation of thyrotoxicosis and consequently intensify the urticaria. In such patients, a trial period of carbimazole‐free therapy in order to closely assess urticarial response would be justified. Talapatra et al^19^ faced the above dilemma in 2007, and opted to withhold carbimazole therapy, resulting in an aggravated thyrotoxicosis with a persistent rash. They then found the urticarial rash to improve once the patient's hyperthyroid state had resolved with propylthiouracil.

In our patient, the hyperthyroidism developed shortly before urticaria, although she was clinically euthyroid when she presented to hospital. But despite the unremarkable FT4 level, it is likely that the IgE anti‐TPO cascade was still rampant as evidenced by the high anti‐TPO levels. Interestingly, the decision to stop carbimazole did result in symptomatic relief; however, this coincided with high dose antihistamines.

Overall, urticaria is a complication of both autoimmune hyperthyroid disease and the medications used to treat hyperthyroidism. Differentiating between the two is both vital and challenging. In the absence of other clues, it is worth attempting a trial period of withholding antithyroid medications with close assessment of the urticarial rash (see Table 2). At the same time, symptomatic relief can be found with steroids and antihistamines.

**TABLE 2:** Summary of recommendations

Thyroid function tests should be part of routine screening for patients presenting with acute or chronic urticaria. In patients that are clinically euthyroid with urticaria or rash, antithyroid drugs can be stopped temporarily. In patients with clinical hyperthyroidism and urticaria, a trial of withholding antithyroid drugs can be utilized with close assessment of response. Short‐term symptomatic relief of urticaria can be found with high dose antihistamines and steroids.

## CONFLICT OF INTEREST

None declared.

## AUTHOR CONTRIBUTION

NW: involved in conception/analysis, drafting of the article, critical revision of the article, and final approval. EJ: involved in conception/analysis, critical revision of the article, and final approval.

## ETHICAL APPROVAL

Hereby, I, Dr Nicholas Womack, consciously assure that for the manuscript “Urticaria as a Manifestation of Hyperthyroidism” the following is fulfilled: (1) This material is the authors' own original work, which has not been previously published elsewhere. (2) The paper is not currently being considered for publication elsewhere. (3) The paper reflects the authors' own research and analysis in a truthful and complete manner. (4) The paper properly credits the meaningful contributions of coauthors and coresearchers. (5) The results are appropriately placed in the context of prior and existing research. (6) All sources used are properly disclosed (correct citation). Literally copying of text must be indicated as such by using quotation marks and giving proper reference. (7) All authors have been personally and actively involved in substantial work leading to the paper, and will take public responsibility for its content.

## Data Availability

The data that support the findings of this study are openly available through Wiley at DOI: https://doi.org/10.22541/au.159112170.04349002.
